# The Final Mile: Evaluating eHealth and mHealth Utilization Among Older Adults

**DOI:** 10.1093/geroni/igab046.995

**Published:** 2021-12-17

**Authors:** Mary Krebs

**Affiliations:** University of Massachusetts Boston, Warren, Maine, United States

## Abstract

While health endures as a term to describe looking after oneself, looking after loved ones, and receiving care, the added component of electronic technology has emerged to affect all levels of health care delivery. Despite the prevalence of digital health and empirical evidence strongly supporting improved outcomes--the final mile--as it is sometimes called, is the sustained patient engagement with eHealth and mHealth we have yet to achieve. This research identifies a gap in the literature for understudied characteristics of digital health adoption, use affecting the aging population in the U.S., and contributes a deeper understanding of the key barriers to use of health-related technology. A mixed-methods research approach explores prevalent barriers to digital health utilization by older adults through a pre-post data collection strategy to empirically test an educational health-related intervention rooted in the Technology Acceptance Model. This validated analytic framework represents a decision ‘core’ as a user pathway for actual use. Evaluation of score data utilized a quantitative test of group means while thematic coding was employed for qualitative analysis. The results from this study are two-fold. The work strongly suggests specific barriers to adoption and use, confirming a distrust and reluctance to engage. However, additional evidence, both quantitative and qualitative illuminates substantive skills, positive perceptions, hopeful attitudes, as well as the rationale for use of available digital resources. Findings suggest future research would benefit from expanded use of the two-pronged approach to foster health-related technology engagement.

